# Durable and High‐Performance Triboelectric Nanogenerator Based on an Inorganic Triboelectric Pair of Diamond‐Like‐Carbon and Glass

**DOI:** 10.1002/advs.202309170

**Published:** 2024-07-01

**Authors:** Wenjian Li, Liqiang Lu, Chi Zhang, Katja Loos, Yutao Pei

**Affiliations:** ^1^ Advanced Production Engineering Engineering and Technology Institute Groningen Faculty of Science and Engineering University of Groningen Nijenborgh 4 Groningen 9747 AG The Netherlands; ^2^ Beijing Institute of Nanoenergy and Nanosystems Chinese Academy of Sciences No. 8, Yangyandong 1st Road, Yanqi Economic Development Zone, Huairou Beijing 101400 China; ^3^ Macromolecular Chemistry & New Polymeric Materials Zernike Institute for Advanced Materials Faculty of Science and Engineering University of Groningen Nijenbogh 4 Groningen 9747AG The Netherlands

**Keywords:** diamond‐like carbon, TENG, triboelectric nanogenerator, ultradurable

## Abstract

The long‐term durability of triboelectric nanogenerators (TENGs) remains a main challenge for practical applications because of inevitable material abrasion and wear, especially for sliding TENGs. Herein, an inorganic triboelectric pair composed of diamond‐like carbon (DLC) and glass with excellent durability and triboelectric output for sliding‐mode TENGs is proposed. This triboelectric pair possesses a low coefficient of friction and little abrasion and accordingly excellent durability (>500 000 cycles). Moreover, compared with the traditional copper‐polytetrafluoroethylene (Cu‐PTFE) TENG with maximum transferred charges of 50 nC, those of the DLC‐glass TENG reaches 141 nC. Due to the low‐friction and high hardness of the triboelectric pair, the output quickly recovers after simply cleaning wear debris. The DLC‐glass TENG demonstrates an output power density of 530 mW m^−2^ and a fourfold faster capacitor charging speed than the Cu‐PTFE TENG. Compared to the reported durable TENGs via structure optimization and interface lubrication, the DLC‐glass TENG shows higher outputs and simpler structure. This DLC‐glass pair structure is also introduced into a spherical TENG for blue energy harvesting with excellent durability. The inorganic triboelectric pair with excellent mechanical durability and electrical performance proposed in this work shows huge prospects for practical applications of TENGs.

## Graphical abstract

An inorganic triboelectric nanogenerator composed of diamond‐like carbon and glass is proposed, exhibiting excellent durability (>500 000 cycles) and triboelectric output (141 nC) in comparison with traditional polymer‐based pairs. The low‐friction and high hardness of the triboelectric pair enables the output quickly recovered after simply cleaning wear debris.

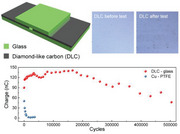

## 1 Introduction

The worsening energy crisis and environmental pollution on earth make it urgent and significant to develop clean and sustainable energy techniques. Moreover, with the flourishing development of the distributed Internet of Things, in which numerous miniaturized electronics are widely scattered for sensing and monitoring, efficient energy harvesting technologies are highly desired to enable those electronics to be self‐powered to relieve the huge replacement cost and potential pollution of depleted batteries.^[^ ^1^ ^]^ The triboelectric nanogenerator (TENG), based on the coupling of contact electrification and electrostatic induction, has been praised as an emerging superior clean energy technique.^[^ ^2^ ^]^ By virtue of the merits of simple working principle, various working modes, universal material choices, high output, high efficiency, easy fabrication and so on, TENGs have shown enormous prospects in many kinds of applications, from self‐powered sensing^[^ ^3^ ^]^ and micro/nano energy harvesting^[^ ^4^ ^]^ to high‐voltage sources^[^ ^5^ ^]^ and large‐scale blue energy harvesting.^[^ ^6^ ^]^ Specifically, sliding‐mode TENGs,^[^ ^7^ ^]^ due to their simpler structure and higher charge transfer efficiency, have been identified as a more efficient energy harvesting technique for practical applications. Regardless of the application kind, the output of the TENG has always been expected to be as high as possible for sensitive sensing or effective energy harvesting. Usually, to reach the maximum output, intimate contact, and friction between the triboelectric materials as well as ultrathin triboelectric layers are necessarily required in TENGs, especially for polymers in sliding‐mode TENGs. However, the wear and tear of the triboelectric pair after long‐term friction can lead to a dramatic output degradation and accelerate the failure of TENGs.

To break through the bottleneck of poor durability of sliding TENGs, many strategies, including structural optimization, interfacial lubrication, and materials innovation, have been proposed. In terms of structural optimization, the noncontact freestanding mode with a small gap between the triboelectric pair,^[^ ^8^ ^]^ rolling friction mode,^[^ ^9^ ^]^ and soft contact mode using soft fur or flexible dielectric strips^[^ ^10^ ^]^ were developed to avoid severe abrasion of triboelectric materials. Nevertheless, the output performance is inevitably sacrificed a lot due to the lack of intimate contact, so additional charge replenishment designs were required,^[^ ^11^ ^]^ which made the structure of TENGs more complex and bulkier. For example, Chen et al. developed an automatic mode transition TENG to mediate the severe abrasion during contact mode, but the output could quickly decreased from 99 to 62 nC when transiting from the contact mode to noncontact mode.^[^ ^12^ ^]^ To overcome the low output of the noncontact TENG, Zhou et al. proposed to inject charges from a lightly contacted TENG to a no‐wear dual‐capacitor enhancement system and achieved an enhanced output, but largely increased the structure complexity of the whole device.^[^ ^8c^ ^]^ Learning from the indispensable lubrication in mechanical engineering, interfacial lubrication has recently become a quite popular method applied to reduce the coefficient of friction (CoF) between the triboelectric pair.^[^ ^13^ ^]^ Surprisingly, interfacial liquid lubrication can also help improve the output of TENGs because dielectric liquid lubricants with high breakdown strength can penetrate into microgaps and voids and suppress interfacial electrical breakdown between materials. However, the optimal lubricant type and dosage are quite different, depending on the triboelectric materials, and the output can gradually decrease with the volatility of lubricants, which are also critical issues in practical applications. For example, Wu et al. showed that adding heptane between the triboelectric pair, the output can increased to 430 V but it quickly decreased to 160 V just after 23 s because of the evaporation of heptane.^[^ ^13a^ ^]^ In addition, some wear‐resistant materials, such as diamond‐like carbon (DLC),^[^ ^14^ ^]^ butylated melamine formaldehyde,^[^ ^15^ ^]^ polyvinyl chloride/molybdenum disulfide composite,^[^ ^16^ ^]^ metallic glass,^[^ ^17^ ^]^ and cold rolled metal,^[^ ^18^ ^]^ were developed to improve durability, whereas TENGs composed of these materials usually had low outputs because of the poor contact electrification performance between the applied triboelectric pairs. For example, Choi and co‐workers used the hydrogenated DLC paired with polytetrafluoroethylene (PTFE), the durability of the TENG is enhanced but the output voltage was quite low (<40 V).^[^ ^14^ ^]^ They further proposed Si‐DLC as a low‐friction material, but its output was still limited even after water lubrication (80 V).^[^ ^19^ ^]^ Therefore, triboelectric pairs that simultaneously possesses the advantages of simple structure excellent mechanical durability and electrical performance are highly desired for sliding‐mode TENGs.

Herein, we propose a ultradurable and high‐performance sliding‐mode TENG based on an inorganic triboelectric pair. The sliding‐mode TENG consists of two DLC electrodes and a freestanding glass slider that reciprocally slides over the two DLC electrodes (DLC‐glass TENG). Owing to the excellent wear resistance, ultrahigh hardness, and ultralow friction of DLC against glass, the CoF between this triboelectric pair was as low as 0.11. As a result, the DLC‐glass TENG suffered minor surface abrasion and achieved an excellent output durability over 500 000 working cycles. In addition, the DLC‐glass TENG showed a high output performance with a maximum short‐circuit transferred charge of 141 nC. In contrast, the transferred charge of the traditional TENG with the most common triboelectric pair of Cu and PTFE pair were 50 nC. Although the output started to decrease due to the inevitable accumulation of wear debris after long‐term operation, it quickly recovered after cleaning the debris. Such an ultrahigh output originated from both the excellent triboelectrification performance and ultrasmooth surfaces between DLC and glass. The DLC‐glass TENG demonstrated an output power density of 530 mW m^−2^ and a fourfold faster capacitor charging speed than the Cu‐PTFE TENG. A spherical TENG based on the DLC‐glass pair was further developed for ocean/water wave energy harvesting. This work proposed an inorganic, ultradurable, and high‐performance triboelectric pair, which is considered to have huge prospects for practical applications of TENGs.

## 2 Results and Discussion

**Figure 1:**
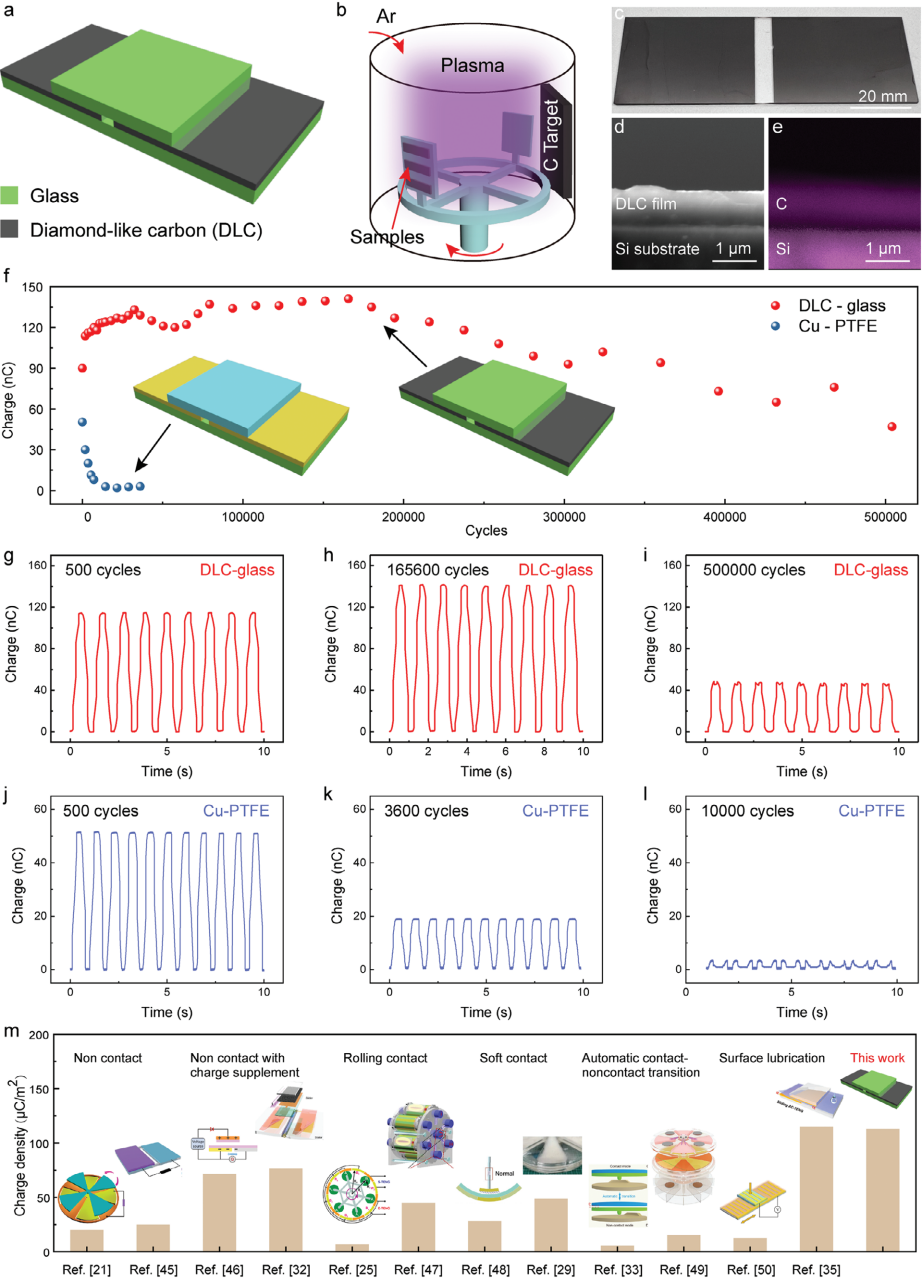
Ultrahigh durability and ultrahigh output performance of the DLC‐glass TENG. a) Schematic structure of the sliding‐mode DLC‐glass TENG. b) Magnetron sputtering deposition of the DLC electrodes. c) Photo of the as‐deposited DLC electrodes on a glass substrate. d) SEM image and e) EDS elemental mapping of the cross‐section view of the deposited DLC. f) Transferred charges of the DLC‐glass TENG and Cu‐PTFE TENG over long‐term testing. g–i) Transferred charge of the DLC‐glass TENG after 500, 165 600, and 500 000 working cycles, respectively. j–l) Transferred charge of the Cu‐PTFE TENG after 500, 3600, and 10 000 working cycles, respectively. m) Comparison of the output charge density of the DLC‐glass TENG with previous works targeted for enhanced durability. Reproduced with permission.^[^ ^8a^ ^]^ Copyright 2014, American Chemical Society. Reproduced with permission.^[^ ^9b^ ^]^ Copyright 2021, American Chemical Society. Reproduced with permission.^[^ ^10c^ ^]^ Copyright 2021, Wiley‐VCH GmbH. Reproduced with permission.^[^ ^11c^ ^]^ Copyright 2022, Wiley‐VCH GmbH. Reproduced with permission.^[^ ^12^ ^]^ Copyright 2020, Wiley‐VCH GmbH. Reproduced with permission.^[^ ^13b^ ^]^ Copyright 2020, Wiley‐VCH GmbH. Reproduced with permission.^[^ ^21a^ ^]^ Copyright 2014, Wiley‐VCH GmbH. Reproduced with permission.^[^ ^21b^ ^]^ Copyright 2021, Springer Nature. Reproduced with permission.^[^ ^21c^ ^]^ Copyright 2018, Elsevier. Reproduced with permission.^[^ ^21d^ ^]^ Copyright 2021, Wiley‐VCH GmbH. Reproduced with permission.^[^ ^21e^ ^]^ Copyright 2022, Wiley‐VCH GmbH. Reproduced with permission.^[^ ^22^ ^]^ Copyright 2021, Elsevier.

The structure of the sliding‐mode DLC‐glass TENG is schematically illustrated in **Figure** **1**a. The DLC‐glass TENG consists of two symmetric DLC electrodes deposited on a glass substrate and one freestanding glass sheet slider that reciprocally slides over the two DLC electrodes. The DLC electrodes were deposited through magnetron sputtering deposition using a graphite target, as shown in Figure 1b. Figure 1c shows a photo of the as‐deposited DLC electrodes on the glass substrate. The thickness of the DLC electrode was ≈0.8 µm, as seen from the scanning electron microscopy (SEM) image of the cross‐section of the DLC coating (Figure 1d) and corresponding energy dispersive spectrum (EDS) elemental mapping (Figure 1e).

Figure S1 (Supporting Information) shows the dimension of the DLC‐glass TENG. The working principle of the DLC‐glass TENG is based on contact electrification and electrostatic induction, as schematically illustrated in Figure S2 (Supporting Information). After several cycles of reciprocating sliding, the glass slider and DLC electrodes were negatively and positively charged, respectively, due to their difference in the ability to gain or lose electrons. When the top glass slid to the position overlapping with the left DLC electrode, negative charges on the glass were electrostatically neutralized with the positive charges on the left DLC electrode (state I). Subsequently, when the top glass gradually slid to the right DLC electrode, the varying potential difference between the two electrodes drives electrons to flow from the right to the left (state II). The electron flow would last until the top glass was fully overlapped with the right electrode (state III). Similarly, when the top glass slid back to the left, electrons flow from the left electrode back to the right electrode (state IV).

As implied by its name, the DLC coating can provide some similar properties to diamond, such as ultrahigh hardness and strong wear resistance, while maintaining an ultralow CoF from graphite.^[^ ^20^ ^]^ In addition, the glass also possesses a relatively high hardness value of ≈5.5 to 7 Mohs. Therefore, the triboelectric pair composed of the DLC and glass is expected to have superior mechanical durability and output stability. The long‐term durability of the sliding‐mode DLC‐glass TENG was first characterized under a load of 5 N and working frequency of 1 Hz. The dynamic short‐circuit transferred charges of the DLC‐glass TENG are demonstrated in Figure 1f. As a comparison, the durability of another traditional TENG with the same size, structure, and similar surface roughness (Figure S3, Supporting Information) using the most common polymer‐based triboelectric pair of Cu and PTFE (Cu‐PTFE TENG) was also investigated. Surprisingly, the DLC‐glass TENG not only demonstrated excellent durability but also ultrahigh output performance in comparison with the Cu‐PTFE TENG (Figure 1f). At the very beginning, the transferred charges of the DLC‐glass TENG were ≈90 nC. Then, the amounts increased sharply to 120 nC after 500 operation cycles (Figure 1g) and thereafter showed a continuous increase to the maximum value of 141 nC after 165 600 cycles (Figure 1h). With long‐time continuous sliding, some debris inevitably formed from the wear of glass, which affected the contact intimacy between the rigid inorganic triboelectric pair. As a result, the transferred charges of the DLC‐glass TENG started to decrease, and finally dropped to 48 nC after 500 000 cycles (Figure 1i). In contrast, the traditional Cu‐PTFE TENG showed an early decrease in the output, rapidly decreasing from 50 nC at 500 cycles (Figure 1j) to 19 nC only after 3600 cycles (Figure 1k). Finally, its output further decreased and only maintained ≈3 nC after 10 000 cycles (Figure 1l). The open‐circuit voltages of the two sliding‐mode TENGs demonstrated a similar trend, as shown in Figure S4 (Supporting Information). This DLC‐glass triboelectric pair not only shows superior output performance than the traditional pairs, such as the Cu‐PTFE pair, but also demonstrates a higher output and simpler structure compared with the previously reported TENGs targeted for enhanced durability via structure optimizations^[^ ^8^, ^9^, ^10^, ^11^, ^12^, ^21^ ^]^ and interface lubrications,^[^ ^13^, ^22^ ^]^ as illustrated in Figure 1m.

Wear debris is inevitable during sliding, but the influence of the debris on the subsequent friction and triboelectric output differs greatly between the two triboelectric pairs. Figure S5 (Supporting Information) demonstrates the measured hardness and modulus of the four materials. The DLC film has an ultrahigh hardness and modulus of 19.9 and 185.8 GPa, respectively. Although compared with DLC, the glass has a decreased hardness and modulus of 6.9 and 97.4 GPa, which still rank it as a hard material. In contrast, the hardness and modulus of Cu and PTFE are much lower. The Cu possesses a hardness and modulus of 1.3 and 126.9 GPa, respectively. Specifically, the PTFE features an extreme low hardness of 0.05 GPa and its modulus is ≈0.8 GPa. As a result, the debris of the DLC‐glass TENG was found almost to be silica microparticles (1–2 µm), while that of the Cu‐PTFE TENG was mainly large PTFE sheet (0.5–2 mm) with some small Cu sheet, as proven by their SEM image and EDS elemental mapping (Figure S6, Supporting Information). In the case of DLC‐glass pair, the silica debris between the interface acted as rigid microballs, which reduced the contact intimacy and effective contact area by creating a microgap between the triboelectric pair; thus, the output started to decrease. For the Cu‐PTFE pair, the debris was mainly soft PTFE sheets that tightly adhered to the surface and significantly reduced effective contact area. Moreover, it is worth noting that the rigid debris on the DLC electrodes can be easily removed, while that on the Cu electrodes is very difficult to clean, which will be discussed later. It is clear that the nonpolymer‐based DLC‐glass triboelectric pair demonstrated a superior output performance as well as output durability over the traditional polymer‐based triboelectric pair.

**Figure 2:**
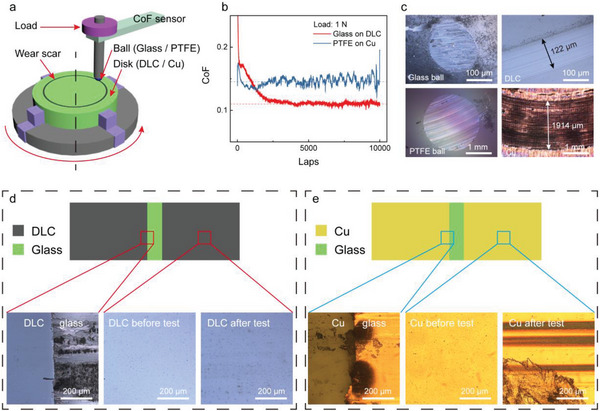
Tribological properties and surface wear of the DLC‐glass triboelectric pair. a) Schematic illustration of the ball‐on‐disk tribological test. b) Tested dynamic CoF of the DLC‐glass tribo‐pair and Cu–PTFE tribo‐pair. c) Wear scars on the balls and coatings after tribo‐test. d) and e) Surface wear analysis of the sliding‐mode DLC‐glass TENG d) and Cu–PTFE TENG e).

The tribological property and surface wear of the DLC‐glass pair were then systematically analyzed. A standard ball‐on‐disk tribological test, as schematically illustrated in **Figure** **2**a, was conducted to measure the dynamic CoF between the DLC‐glass pair during long‐term sliding. Figure 2b shows the measured CoF of the DLC‐glass tribo‐pair, as well as the Cu‐PTFE tribo‐pair over 10 000 laps under a load of 1 N. It is worth noting that due to the difference in the ball diameter and material modulus, the actual contact pressure between the DLC‐glass and Cu‐PTFE tribo‐pair was significantly different. The maximum contact pressure between the DLC‐glass tribo‐pair was calculated as 404.3 MPa, while that between the Cu‐PTFE tribo‐pair was only 15.2 MPa. At the initial stage, the CoF between the DLC‐glass pair was higher than that of the Cu‐PTFE pair. The reason is that for the DLC‐glass pair, the debris first to appear was glass microparticles, while that of the Cu‐PTFE pair was PTFE sheet. PTFE itself is a widely used solid lubricant, so the initial CoF of the Cu‐PTFE pair could be lower than DLC‐glass pair. Thereafter, DLC and Cu debris started to appear, but in contrast, the DLC debris served as an interface solid lubricant due to its low CoF. As a result, the CoF between the DLC‐glass pair gradually decreased while that of the Cu‐PTFE pair increased, and both finally entered the dynamically steady states. In the steady state, the CoFs between the DLC‐glass pair and Cu‐PTFE pair were 0.11 and 0.15, respectively. The DLC‐glass pair showed a much lower CoF than Cu‐PTFE pair even though its contact pressure was 27 times higher than that of Cu‐PTFE, revealing the excellent lubricity and long‐term durability of DLC. The CoF between other polymer‐based triboelectric pairs can even be higher since PTFE is already a solid lubricant. The wear scars on the balls and coatings were investigated under an optical microscope and are shown in Figure 2c. The width of the wear scar on the Cu‐PTFE pair was ≈1914 µm, which was 15 times larger than that of the DLC coating and glass ball (122 µm), implying the excellent wear resistance of the DLC‐glass pair. EDS elemental mapping of the scars on the balls (Figure S7, Supporting Information) after the tribological test shows that the debris of the DLC‐glass pair was mainly from glass, as there was rarely carbon detected on the glass ball, while a significant amount of Cu was transferred to the PTFE ball. The wear rates of the DLC electrode and Cu electrode are shown in Figure S8 (Supporting Information), showing that the wear rate of the DLC is ≈562 times smaller than that of Cu.

The mechanical abrasion of the DLC‐glass TENG was then investigated. Figure 2d,e shows the surface wear analysis of the DLC and Cu electrodes of the two sliding‐mode TENGs after their long‐term durability tests, respectively. Note that the number of operation cycles for the DLC‐glass TENG was 500 000, while that of the Cu‐PTFE TENG was only 10 000. Due to the ultrahigh hardness and low CoF of the DLC, the DLC electrodes showed no obvious scratches and abrasions after the test. Specifically, without the protection of the DLC film, the glass substrate exposed in the gap between the two DLC electrodes faced severe abrasion. Only some tiny peelings could be found on the sharp edge of the DLC electrodes. It was the sharp edge that led to some inevitable abrasions on the top glass slider (Figure S9a, Supporting Information). In the case of the traditional Cu electrodes, there were obvious severe abrasions on both its edge and center. In contrast, the glass substrate was essentially free from abrasion due to its much higher hardness than Cu tape and PTFE. In addition, severe wear scars could also be obviously observed on the PTFE slider (Figure S9b, Supporting Information), even though PTFE can serve as a solid lubricant during friction. This also implies that the debris in the DLC‐glass pair almost came from the glass, while that in the Cu‐PTFE pair was from both the Cu and PTFE. Figure S10 (Supporting Information) shows optical photos of the stators (electrode part) of the two sliding‐mode TENGs after the long‐term durability test, from that one can see that the DLC electrodes were still glossy and optically reflective, while the Cu electrodes were no longer shiny because of abrasion and debris adhesion. The above results clearly show the superior mechanical durability of the DLC and glass triboelectric pair.

**Figure 3:**
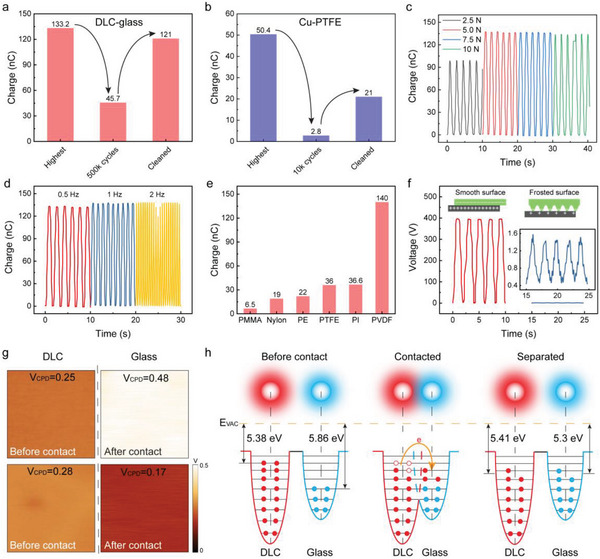
Electrical characteristics of the sliding‐mode TENGs. a) Output recovery of the DLC‐glass TENG after cleaning the debris. b) Output recovery of the Cu‐PTFE TENG after cleaning the debris. c) and d) Output response of the DLC‐glass TENG under different loads (c) and different sliding frequencies d). e) Outputs of the DLC TENG with different counterpart materials as the slider. f) Output of the DLC‐glass TENG with frosted glass as the freestanding slider. g) KPFM surface potential measurements of DLC and glass before and after contact with each other. h) Surface state‐electrode cloud overlap model of the contact electrification between DLC and glass.

Since the DLC and glass both possess a high hardness, the debris generated was almost rigid microparticles shedding from the glass slider, and it is difficult for the rigid debris to adhere back to the friction interface. Even though the accumulated debris could gradually decrease the output performance because of the reduced contact intimacy and effective contact area, such debris was very easy to fully clean. As a result, the output of the DLC‐glass TENG could recover to almost 90% of its best performance after simple wiping with ethanol, as shown in **Figure** **3**a. The small decrease in the output was due to the abrasion on the glass slider, which slightly decreased the effective contact area between the triboelectric interface. In contrast, the debris from the Cu‐PTFE pair was mainly soft sheets, which were tightly adhered onto the Cu and PTFE surfaces and difficult to fully remove. Therefore, the output performance of the Cu‐PTFE TENG could only be slightly recovered after same cleaning due to the largely decreased effective contact area (Figure 3b).

The output response of the DLC‐glass TENG under different loads was investigated and is shown in Figure 3c. When the load increased from 2.5 to 5 N, the transferred charge accordingly increased from 99 to 137 nC due to the increased contact intimacy. As the DLC and glass were both rigid materials with ultrasmooth surfaces, the contact at 5 N already reached the maximum, and therefore, the output remained stable when the load was further increased to 10 N. A similar trend can be found in the open‐circuit voltage and short‐circuit current (Figure S11, Supporting Information) of the DLC‐glass TENG. The output performance of the DLC‐glass TENG under different sliding frequencies was also investigated. Figure 3d shows the transferred charge with varying frequencies, and the open‐circuit voltage and short‐circuit current are demonstrated in Figure S12 (Supporting Information). The voltage and charge remained constant at different sliding frequencies because the maximum effective contact area was kept constant regardless of the sliding frequency. The current increased linearly with increasing frequency since the charge transfer rate between the two electrodes increased linearly.

The reason behind the ultrahigh output performance can be attributed to the excellent intrinsic triboelectrification performance and ultrasmooth surfaces between the DLC and glass. To demonstrate the excellent intrinsic triboelectrification performance between DLC and glass, some of the most commonly used polymers (PMMA, nylon, PE, PTFE, PI, and PVDF) in TENGs with strong triboelectric positivity or negativity were adopted to replace the top glass slider. As shown in Figure 3e, most polymer sliders showed a low output with the DLC electrodes, except PVDF, which exhibited comparable outputs with the glass. PMMA, Nylon, and PVDF coatings were prepared by spin coating method and thus are likely to have similar surface roughness, while PTFE, PI, and PVDF possess similar triboelectric negativity. This proves that the ultrahigh output performance of the DLC‐glass triboelectric pair originally came from the excellent intrinsic triboelectrification. Besides, hydrogenated DLC (H‐DLC) with a lower CoF was used to replace the pure DLC to prove the importance of intrinsic triboelectrification. As shown in Figure S13 (Supporting Information), although the CoF of H‐DLC is lower than that of DLC, the H‐DLC‐glass TENG exhibited a lower triboelectric output because of the increased electric resistance of H‐DLC that affected the triboelectrification effect. In traditional polymer‐based TENGs, microstructures and surface roughness can enhance the output performance because soft materials can conformally deform under pressure and the effective contact area can be enhanced by the intimately contacted microstructures.^[^ ^23^ ^]^ For rigid materials with high hardness, surface microstructures are unable to deform largely but remain point contacts at microscale asperities, leading to a significant decrease in the effective contact area. As shown in Figure 3f, the output of the sliding‐mode DLC‐glass TENG using forested glass as the top slider was extremely low, showing an open‐circuit voltage of only 1.6 V. Figure S14 (Supporting Information) shows the SEM image of the large surface roughness of the frosted glass slider. It is clear that the ultrasmooth surfaces of DLC and glass are also essential to ultrahigh output performance. Surface potential measurement of DLC and glass before and after sliding, using Kelvin probe force microscopy (KPFM), directly reveals the surface charge transfer between them. As shown in Figure 3g, before contact with each other, DLC and glass show a contact potential difference (*V*_CPD_) with the KPFM scan tip of 0.25 and 0.48 V, respectively. The work function of the tip is measured as 5.13 eV using an Au sample (5.2 eV). As a result, the work functions of DLC and glass are calculated as 5.38 and 5.86 eV, respectively. In the case of glass, the highest surface state is used to replace the work function as it is a dielectric material. Since the highest surface state of glass is much higher than the Fermi level of DLC, electrons flow from DLC to glass until the highest surface state, and Fermi level reach an equilibrium, resulting in negative charges on the glass surface and positive charges on the DLC surface. Therefore, the highest surface state of glass decreases to 5.3 eV (*V*_CPD_ = 0.17 V) after contacted with DLC, which is similar to the Fermi level of DLC. The contact‐electrification between DLC and glass can be well explained with the surface state‐electrode cloud overlap model (Figure 3h).

**Figure 4:**
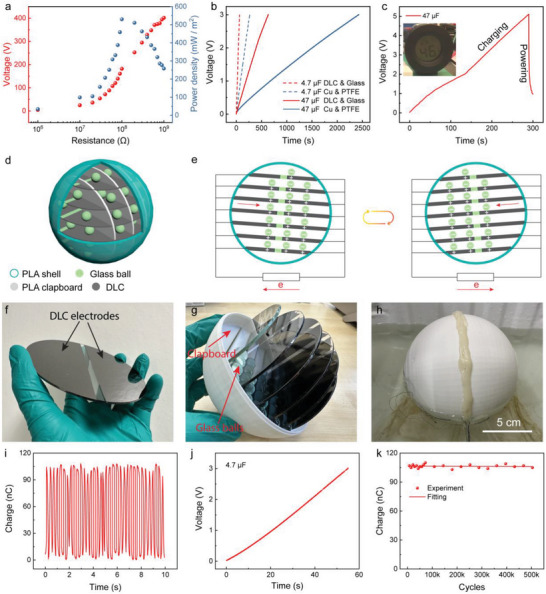
Demonstration of the sliding‐mode DLC‐glass TENG as a practical power source. a) Instantaneous output power density of the DLC‐glass TENG. b) Charging capacitors by the sliding‐mode TENGs. c) Powering a hygrometer by the DLC‐glass TENG. d) Schematic structure and e) working principle of the spherical DLC‐glass TENG. f–h) Photos of the circular electrode plate, spherical DLC‐glass TENG, and the sealed spherical TENG floating on water. i) Transferred charge of the spherical TENG. j) Charging a 4.7 µF capacitor by the spherical TENG. k) Output stability of the spherical TENG over 500 000 operation cycles.

With such excellent mechanical durability and electrical performance, the DLC‐glass triboelectric pair can be considered to have huge prospects in micronano energy harvesting as practical power sources. First, the output power density of the DLC‐glass TENG was characterized through impedance matching. As demonstrated in **Figure** **4**a, the maximum instantaneous power density of the sliding‐mode DLC‐glass TENG reached 530 mW m^−2^ at an external resistance of 100 MΩ, which also indicates that the internal impedance of the DLC‐glass TENG is 100 MΩ. Figure 4b shows the voltage curves of two capacitors, with capacitances of 4.7 and 47 µF, respectively, charged by the two sliding‐mode TENGs under a sliding frequency of 1 Hz through a full‐wave rectifier. The charging speed of the DLC‐glass TENG was almost 4 times as fast as that of the Cu‐PTFE TENG. Taking the 4.7 µF capacitor as an example, the DLC‐glass TENG took 63 s to charge the capacitor to 3 V, while the Cu‐PTFE TENG took 265 s. A hygrometer was powered by the DLC‐glass TENG to demonstrate its prospects as a practical power source for low‐power consumption electronics (Figure 4c; Video S1, Supporting Information).

Furthermore, a spherical DLC‐glass TENG was developed based on the ultradurable and ultrahigh performance DLC‐glass triboelectric pair to harvest the random and low‐frequency energy of ocean/water waves. The structure of the spherical DLC‐glass TENG is schematically illustrated in Figure 4d. Multi‐layer electrode plates were sealed in a spherical shell (120 mm in diameter) and the electrodes from each side were connected together. Clapboards were used to split the electrode plate into fixed sliding channels for a group of glass balls (10 mm in diameter). Figure 4e shows the working principle of the spherical DLC‐glass TENG. Under the excitation of random ocean/water waves, the glass balls roll randomly over the DLC electrodes and reciprocates across the gap between the two DLC electrodes, driving electrons flow back, and forth between the two electrodes. Figure 4f–h show optical photos of the as‐fabricated circular electrode plate, spherical DLC‐glass TENG and the sealed spherical TENG floating on water. To characterize its wave energy harvesting performance, the spherical DLC‐glass TENG was fixed on a linear motor with a reciprocating displacement of 3 cm and frequency of 2 Hz to simulate the water wave. Figure 4i shows the short‐circuit transferred charge of the spherical DLC‐glass TENG, which was ≈105 nC. The output voltage and current of the spherical TENG are demonstrated in Figure S15 (Supporting Information). With such high output, it can charge a 4.7 µF capacitor to 3 V in 55 s, revealing its potential in macroscale blue energy harvesting (Figure 4j). More importantly, the friction between the ball and DLC electrodes changed to rolling friction, which can further significantly decrease the abrasion and enhance the long‐term durability, as proven by the stable output over 500000 cycles (Figure 4k).

## 3 Conclusion

An inorganic, ultradurable, and high‐performance triboelectric pair based on DLC and glass was proposed for sliding‐mode TENGs. Owing to the excellent wear resistance, ultrahigh hardness, and low friction of DLC electrodes against glass sliders, the DLC‐glass TENG exhibited excellent durability compared with the traditional polymer‐based Cu‐PTEF TENG. In addition to mechanical durability, the DLC‐glass TENG also showed ultrahigh electrical performance. The maximum short‐circuit transferred charge reached 141 nC, while that of the Cu‐PTEF TENG was just 50 nC. The ultrahigh output of the sliding‐mode DLC‐glass TENG originated from both the intrinsic strong triboelectrification performance and ultrasmooth surfaces between the DLC electrodes and glass slider. Although debris inevitably began to accumulate after hundreds of thousands of cycles and led to a decrease in the output, the output could be quickly recovered after cleaning the debris. Micronano energy could be efficiently harvested by the DLC‐glass sliding TENG, and electronics, such as a hygrometer, could be easily powered. A spherical DLC‐glass TENG was further developed based on the DLC‐glass triboelectric pair for blue energy harvesting. This work proposed an ultradurable, ultrahigh performance, and nonpolymer‐based triboelectric pair, demonstrating huge prospects toward practical applications of energy harvesting.

## 4 Experimental Section

### Fabrication of the Sliding Triboelectric Nanogenerator (TENG)

Microscopy glass slides (75 × 25 × 1 mm) were selected as the substrate for depositing DLC and Cu electrodes. Before deposition, the glass slide was ultrasonically cleaned first in acetone for 15 min and then ethanol for another 15 min. The center of the cleaned glass slide was covered by high‐temperature resistive tape (width: 5 mm) to create a gap between the two electrodes. Electrodes were deposited by magnetron sputtering deposition using a Teer UDP400 magnetron sputtering system. A pulsed DC power unit (Pinnacle plus, Advanced Energy) was used for the substrate bias source, operating at 250 kHz and 87.5% duty cycle for plasma cleaning and deposition. The flow rate of Ar was set at 15 sccm, and the sample hold was set to rotate at a constant speed of 3 rpm. During deposition, the glass substrate was first cleaned by Ar plasma with a bias voltage of 300 V for 30 min. Then, a Ti interlayer was deposited on the glass substrate to increase the adhesion between the glass substrate and electrodes using a Ti target in DC power mode with a bias voltage of 40 V and current of 1.5 A for 30 min. Finally, the DLC and Cu electrodes were deposited using a carbon and copper target, respectively, with a bias voltage of 100 V and current of 0.8 A for 3 h. After deposition, the sample was cooled for at least 4 h in the sputtering machine before removal. Hydrogenated DLC was fabricated with the same procedure except that acetylene was introduced into the chamber with a flow rate of 2.5 sccm during the deposition of carbon. For DLC‐glass TENG, another glass sheet (25 × 25 mm) was placed onto the as‐deposited DLC electrodes as the top slider of the sliding‐mode DLC‐glass TENG. For Cu‐PTFE TENG, PTFE slider was prepared by first polishing the surface of a PTFE sheet (25 × 25 × 1.2 mm) and then spin coated with a thin PTFE layer (2% w/v Teflon AF1600 in FC‐70) to further reduce the surface roughness. The final thickness of the PTFE slider is 1 mm, and the polished side was placed onto the Cu electrodes as the top slider.

### Characterization

The tribological test was carried out at room temperature (20–23 °C) and room humidity (≈50%) on a CSM tribometer with a ball‐on‐disc configuration. For the DLC and glass pair, the glass substrate deposited with DLC film was cut into small pieces (25 × 25 mm), which were then glued onto polished M2 steel discs with a diameter of 30 mm, and the counterpart was a commercial glass ball with a diameter of 6 mm. For the Cu and PTFE pair, the glass substrate deposited with Cu coating was glued onto the polished M2 steel discs with similar procedure, and the counterpart was a commercial PTFE ball with a diameter of 13 mm. All the tribo‐tests were carried out at a sliding velocity of 10 cm s^−1^. The hardness and modulus of the materials were measured on a MTS Nano‐indenter XP. The contact pressure between the ball and disk was calculated in CSM InstrumX software (CSM Instruments) by using the modulus measured above. Scanning electron microscopy (SEM) was performed with a Lyra dual beam microscope (Tescan, Czech Republic). Surface wear analysis of the sliding TENGs was investigated by optical microscopy (Olympus digital microscope). The surface roughness and surface potential of the triboelectric materials were measured through atomic force microscopy (AFM) and Kelvin probe force microscopy, respectively, conducted on a Bruker Multimode 8 AFM system under ambient conditions. The electrical output of the sliding‐mode TENGs was measured using a Keithley electrometer (model 6514). A homemade linear motor was applied to provide a reciprocating sliding movement with adjusted frequency and displacement. The stator with electrodes of the TENG was fixed on a height‐adjustable stage, and the top slider was attached to a linear motor. The contact force between the two glasses was measured by a load cell (Chino Sensor, ZNLBM‐5KG).

## Conflict of Interest

The authors declare no conflict of interest.

## Supporting information

Supporting Information

Supplemental Video1

## Data Availability

The data that support the findings of this study are available in the supplementary material of this article.

## References

[1] a) Z. L. Wang , Nano Energy 2019, 58, 669;

[2] a) C. Wu , A. C. Wang , W. Ding , H. Guo , Z. L. Wang , Adv. Energy Mater. 2019, 9, 1802906;

[3] a) Y. Zhou , M. Shen , X. Cui , Y. Shao , L. Li , Y. Zhang , Nano Energy 2021, 84, 105887; 10.3762/bjnano.12.54 PMC8275872 34327113

[4] a) J. Tian , X. Chen , Z. L. Wang , Nanotechnology 2020, 31, 242001; 32092711 10.1088/1361-6528/ab793e

[5] a) J. Wang , Y. Zi , S. Li , X. Chen , MRS Energy Sustain. 2020, 6, E17;

[6] a) Z. L. Wang , T. Jiang , L. Xu , Nano Energy 2017, 39, 9;

[7] S. Niu , Y. Liu , S. Wang , L. Lin , Y. S. Zhou , Y. Hu , Z. L. Wang , Adv. Mater. 2013, 25, 6184. 24038597 10.1002/adma.201302808

[8] a) L. Lin , S. Wang , S. Niu , C. Liu , Y. Xie , Z. L. Wang , ACS Appl. Mater. Interfaces 2014, 6, 3031; 24467654 10.1021/am405637s

[9] a) H. Guo , J. Chen , M.‐H. Yeh , X. Fan , Z. Wen , Z. Li , C. Hu , Z. L. Wan , ACS Nano 2015, 9, 5577; 25965297 10.1021/acsnano.5b01830

[10] a) P. Chen , J. An , S. Shu , R. Cheng , J. Nie , T. Jiang , Z. L. Wang , Adv. Energy Mater. 2021, 11, 2003066;

[11] a) M.‐H. Yeh , H. Guo , L. Lin , Z. Wen , Z. Li , C. Hu , Z. L. Wang , Adv. Funct. Mater. 2016, 26, 1054;

[12] J. Chen , H. Guo , C. Hu , Z. L. Wang , Adv. Energy Mater. 2020, 10, 2000886.

[13] a) J. Wu , Y. Xi , Y. Shi , Nano Energy 2020, 72, 104659;

[14] S. H. Ramaswamy , J. Shimizu , W. Chen , R. Kondo , J. Choi , Nano Energy 2019, 60, 875.

[15] S. S. Kwak , S. M. Kim , H. Ryu , J. Kim , U. Khan , H.‐J. Yoon , Y. H. Jeong , S.‐W. Kim , Energy Environ. Sci. 2019, 12, 3156.

[16] K. Zhao , W. Sun , X. Zhang , J. Meng , M. Zhong , L. Qiang , M.‐J. Liu , B.‐N. Gu , C.‐C. Chung , M. Liu , F. Yu , Y.‐L. Chueh , Nano Energy 2022, 91, 106649.

[17] X. Xia , Z. Zhou , Y. Shang , Y. Yang , Y. Zi , Nat. Commun. 2023, 14, 1023. 36823296 10.1038/s41467-023-36675-x PMC9950355

[18] Y. Ra , J. H. Choi , S.‐J. Choi , M. La , S. J. Park , M.‐J. Kim , D. Choi , Extreme Mech. Lett. 2020, 40, 100910.

[19] M. Gao , S.‐B. Kim , Y. Li , S. H. Ramaswamy , J. Choi , Nano Energy 2023, 105, 107997.

[20] A. Grill , Diamond Relat. Mater. 1999, 8, 428.

[21] a) S. Wang , Y. Xie , S. Niu , L. Lin , Z. L. Wang , Adv. Mater. 2014, 26, 2818; 24449058 10.1002/adma.201305303

[22] K. Wang , J. Li , J. Li , C. Wu , S. Yi , Y. Liu , J. Luo , Nano Energy 2021, 87, 106198.

[23] Y. Zhou , W. Deng , J. Xu , J. Chen , Cell Rep. Phys. Sci. 2020, 1, 100142.

