# Supplementary material for: Durable and High‐Performance Triboelectric Nanogenerator Based on an Inorganic Triboelectric Pair of Diamond‐Like‐Carbon and Glass
Source: Adv Sci (Weinh). 2024 Jul 1;11(33):2309170. doi: 10.1002/advs.202309170 (PMC11434241; doi:10.1002/advs.202309170)
Supplement: Supplementary file 1 — Supporting Information [file ADVS-11-2309170-s002.pdf]

ADVANCED SCIENCE
Open Access

Supporting Information

for *Adv. Sci.*, DOI 10.1002/advs.202309170

Durable and High-Performance Triboelectric Nanogenerator Based on an Inorganic Triboelectric Pair of Diamond-Like-Carbon and Glass

*Wenjian Li, Liqiang Lu, Chi Zhang, Katja Loos and Yutao Pei**

Supporting Information

Durable and High-performance Triboelectric Nanogenerator Based on an Inorganic Triboelectric Pair of Diamond-like-carbon and Glass

*Wenjian Li, Liqiang Lu, Chi Zhang, Katja Loos, Yutao Pei**

W.J. Li., L.Q. Lu, Y.T. Pei

Advanced Production Engineering, Engineering and Technology Institute Groningen, Faculty of Science and Engineering, University of Groningen, Nijenborgh 4, 9747 AG Groningen, the Netherlands

E-mail address: y.pei@rug.nl

C. Zhang

Beijing Institute of Nanoenergy and Nanosystems, Chinese Academy of Sciences, No. 8, Yangyandong 1st Road, Yanqi Economic Development Zone, Huairou District, Beijing 101400, China

K. Loos

Macromolecular Chemistry & New Polymeric Materials, Zernike Institute for Advanced Materials, Faculty of Science and Engineering, University of Groningen, Nijenbogh 4, 9747AG, Groningen, the Netherlands

* E-mail address: y.pei@rug.nl

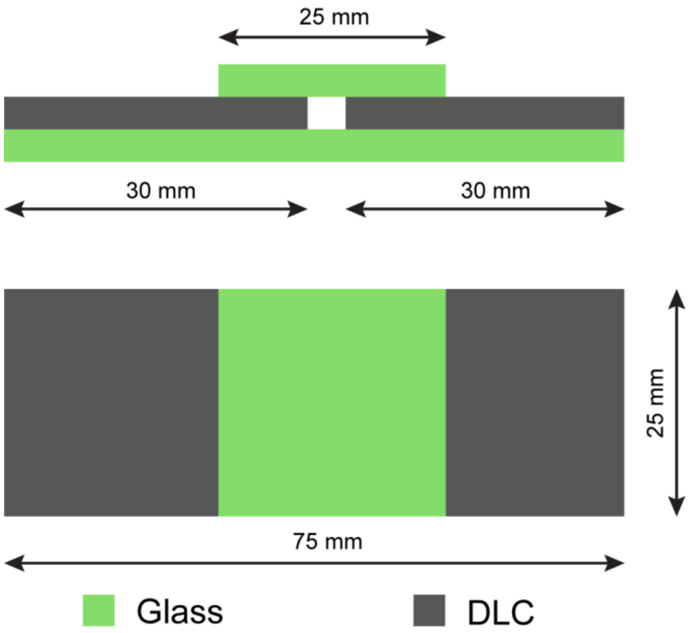

Figure S1. Dimension of the sliding-mode TENG.

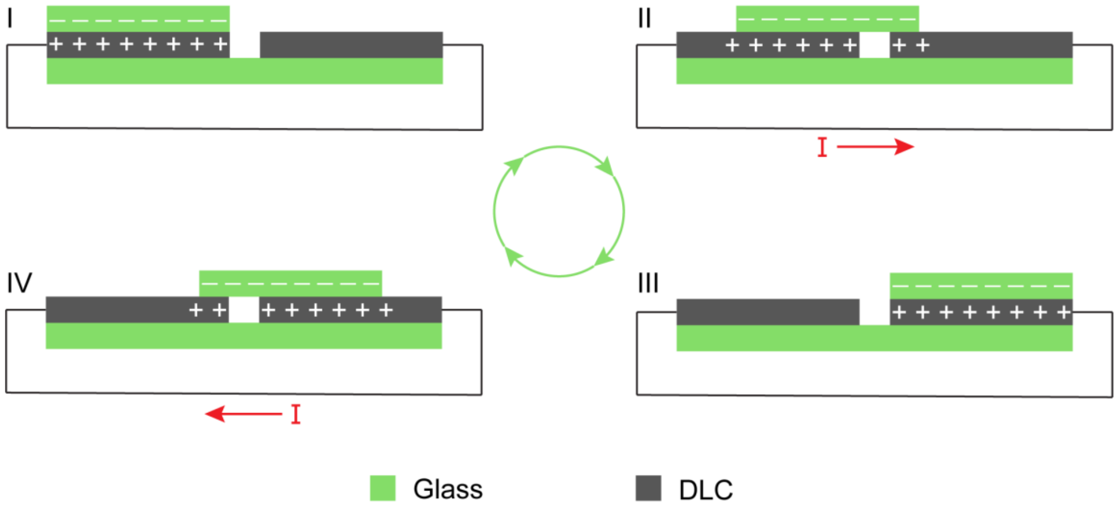

Figure S2. Working principle of the sliding-mode TENG.

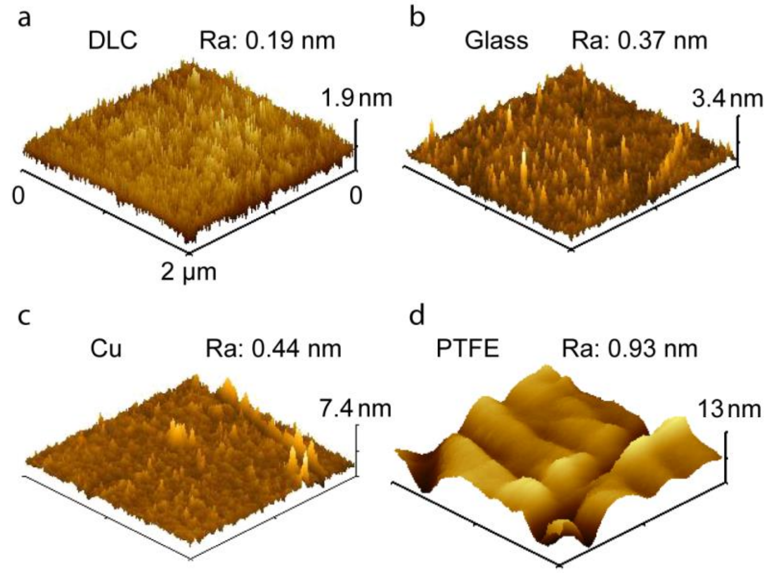

Figure S3. Surface roughness of the DLC, glass, Cu and PTFE.

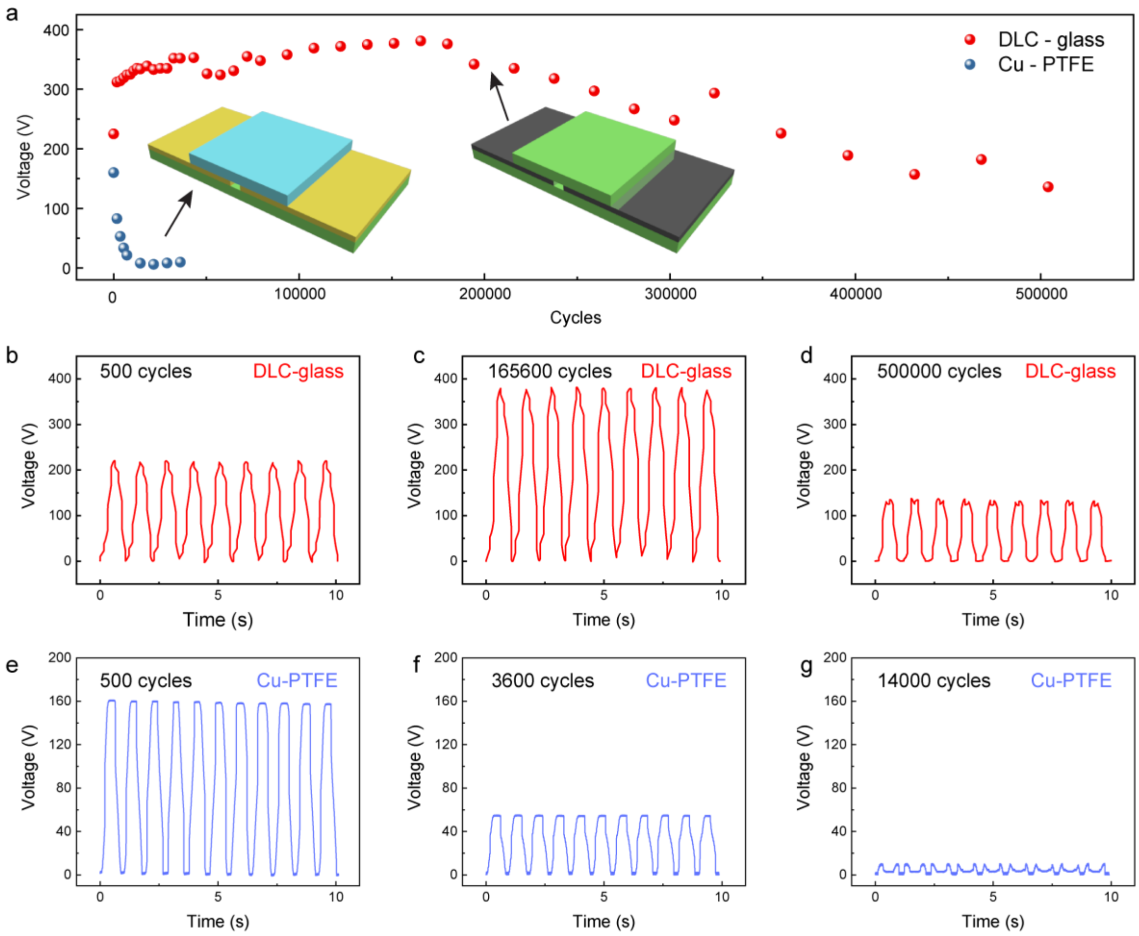

Figure S4. Comparison of the open-circuit voltage outputs of the DLC-glass TENG and Cu-PTFE TENG over long-term durability test. **a)** Open-circuit voltages of the TENGs with different triboelectric pairs: DLC with glass and Cu with PTFE, over long-time test. **b-d)** Transferred charge of the TENG with the pair of DLC and glass after 500, 165600 and 500000 working cycles, respectively. **e-g)** Transferred charge of the TENG with the pair of Cu and PTFE after 500, 3600, 10000 working cycles, respectively.

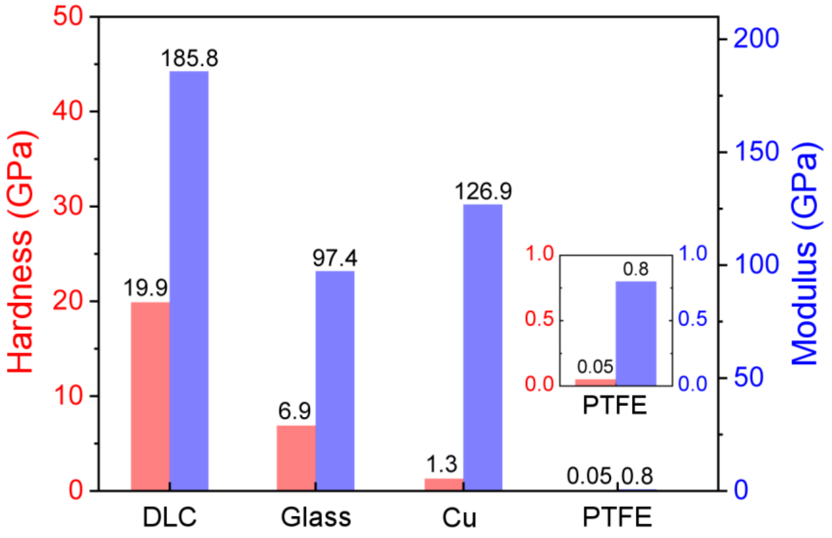

Figure S5. Hardness and modulus of the DLC, glass, Cu and PTFE.

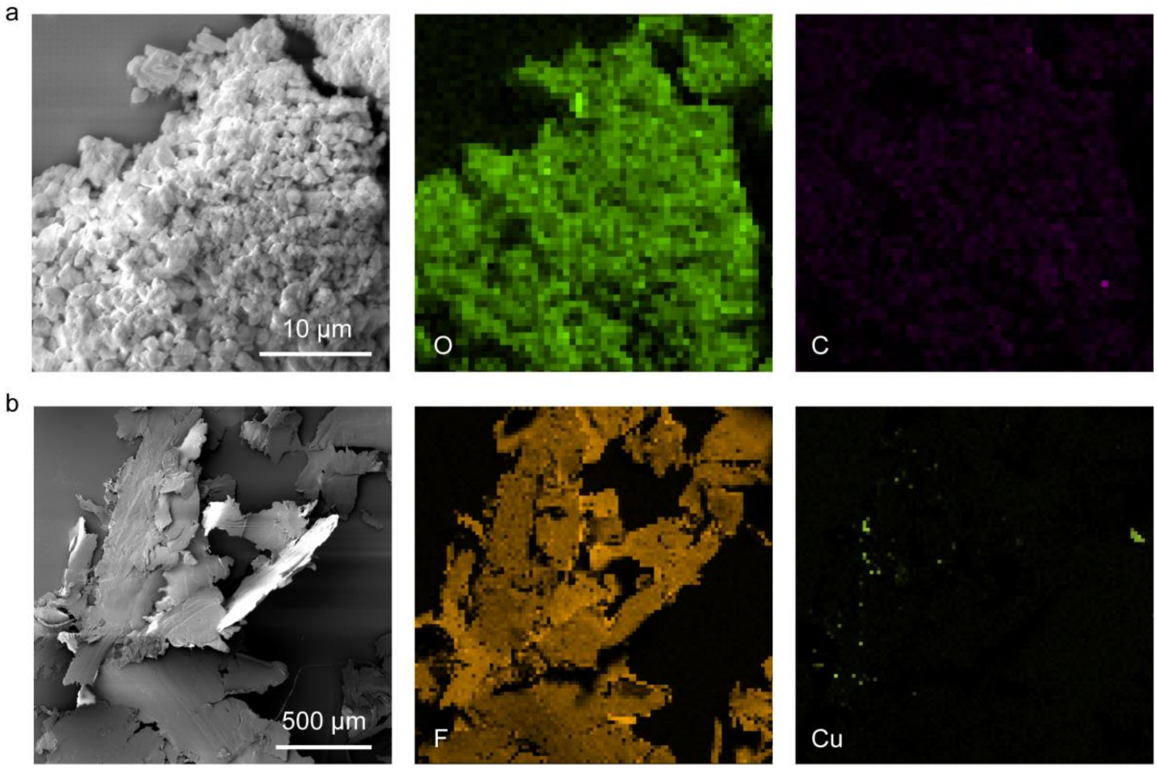

Figure S6. SEM images and EDS elemental mapping of debris formed in the DLC-glass TENG **(a)** and Cu-PTFE TENG **(b)**.

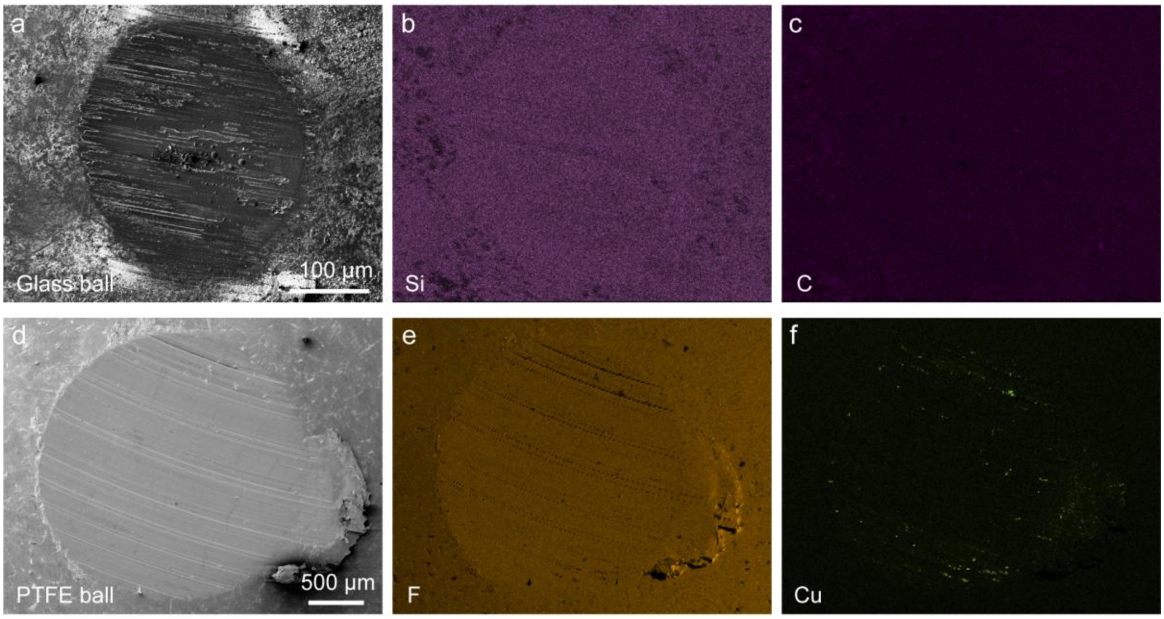

Figure S7. SEM image and EDS elemental mapping of the glass and PTFE ball after tribological testing. SEM image **(a)** and EDS mapping of the element Si **(b)** and C **(c)** of the glass ball. SEM image **(d)** and EDS mapping of the element F **(e)** and Cu **(f)** of the PTFE ball.

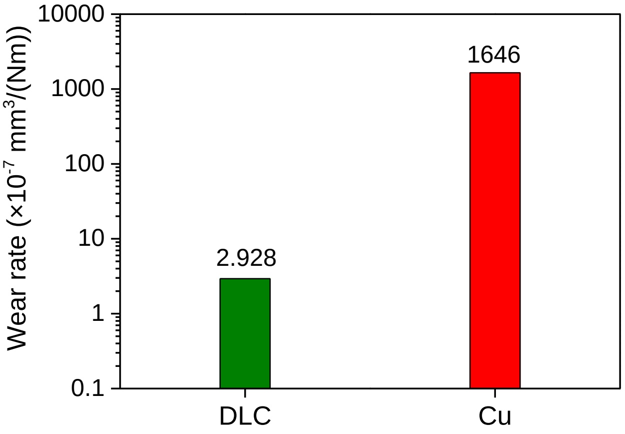

Figure S8. Wear rates of the DLC and Cu electrodes after the tribotest.

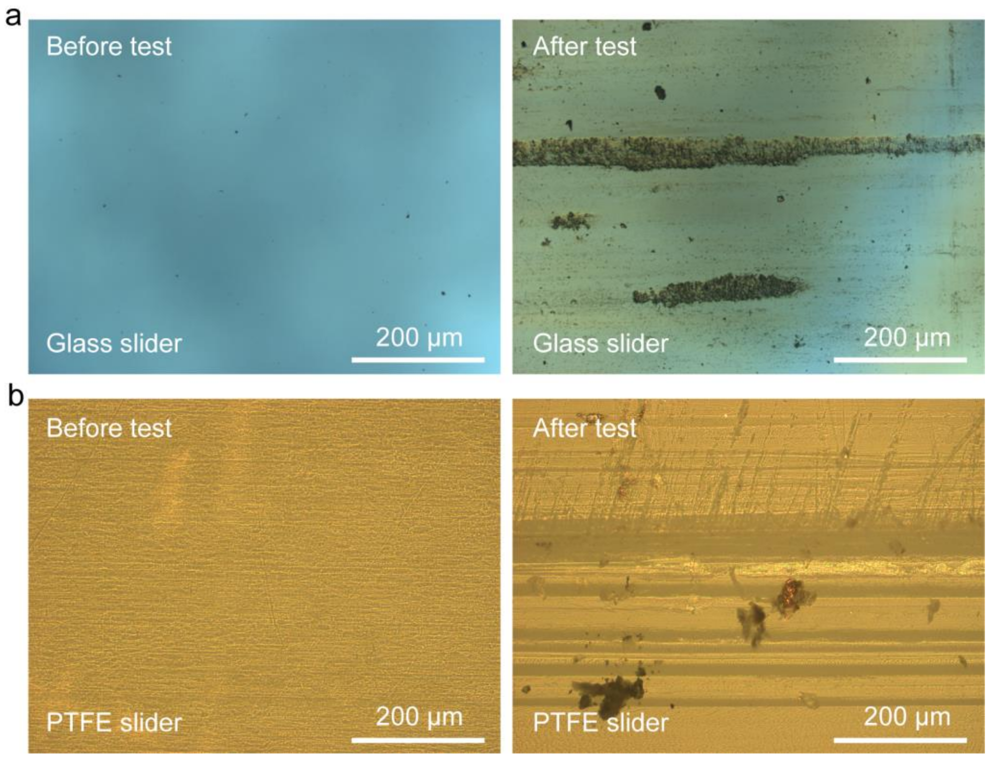

Figure S9. Wear scars on the sliders. **a)** Wear scars on the glass slider after the long-term durability test (500 000 cycles). **b)** Wear scars on the PTFE slider after the long-term durability test (10 000 cycles).

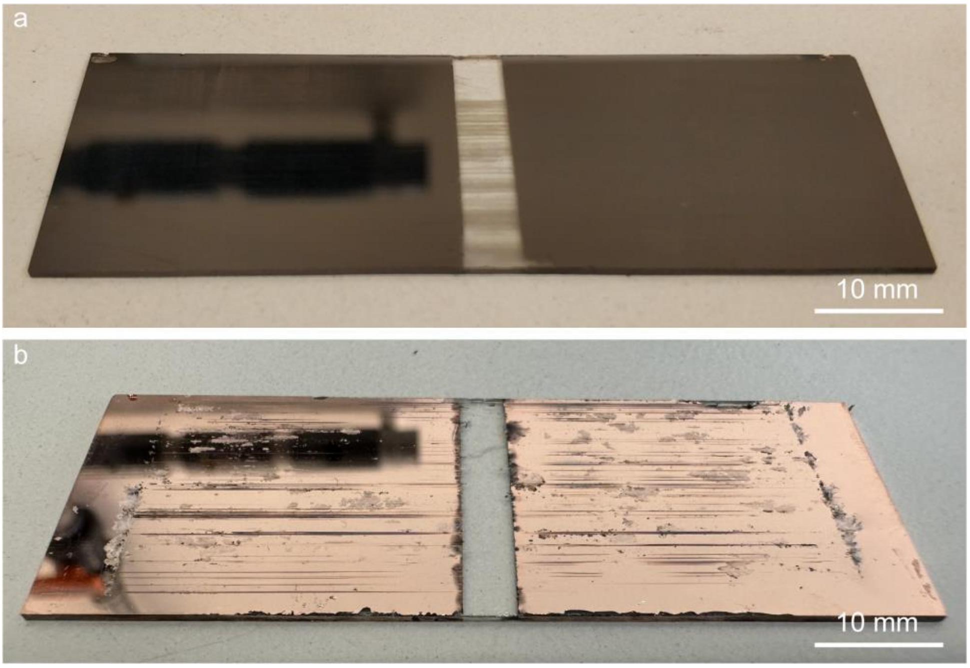

Figure S10. **a)** Optical photo of the stator of the DLC-based sliding TENG after a long-term durability test (500 000 cycles). **b)** Optical photo of the stator of the Cu-based sliding TENG after a long-term durability test (10 000 cycles).

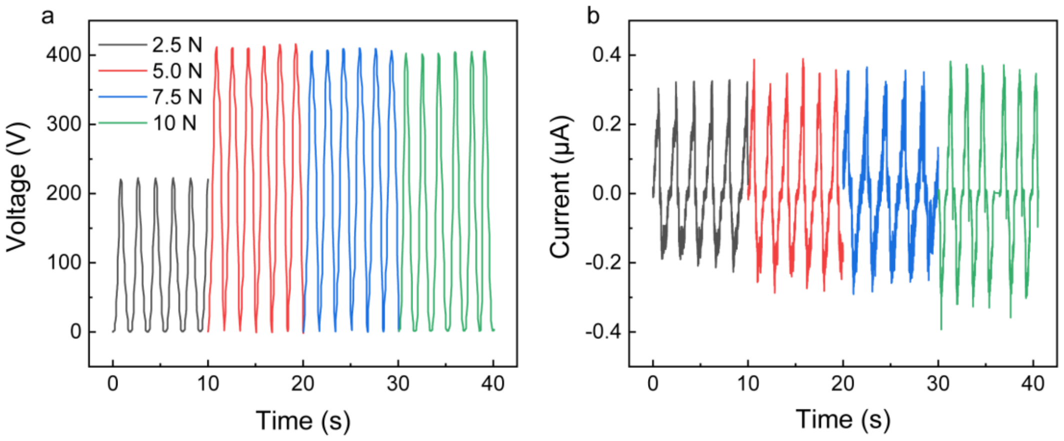

Figure S11. Output voltage and current of the DLC-glass TENG under different loads. **a)** Output voltage and **b)** current of the DLC-glass sliding TENG under different loads indicated.

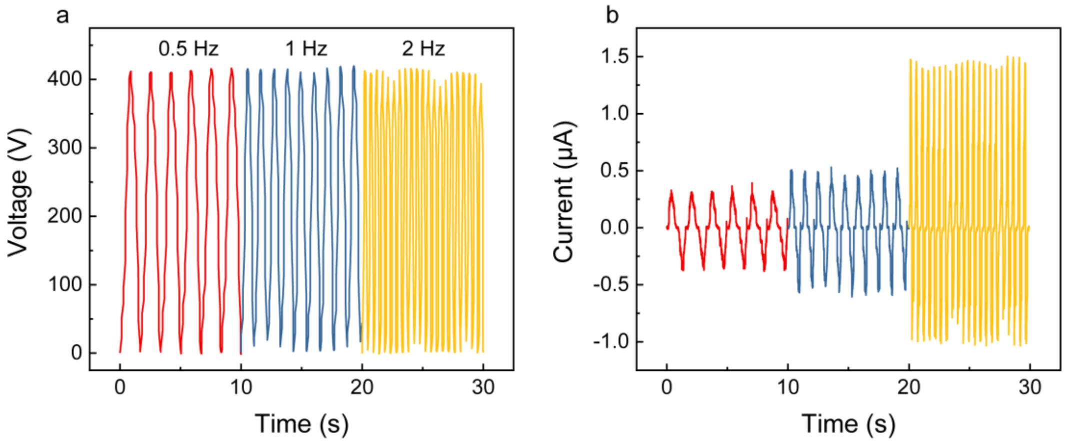

Figure S12. Output voltage and current of the DLC-glass TENG under different frequency. **a)** Output voltage and **b)** current of the DLC-glass sliding TENG under different sliding frequencies indicated.

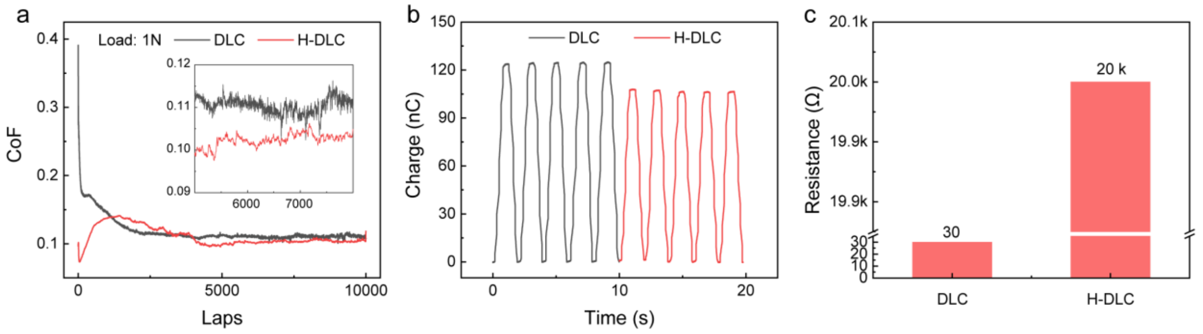

Figure S13. Comparison of the CoF **(a)**, triboelectric output **(b)** and electric resistance **(c)** of H-DLC and DLC.

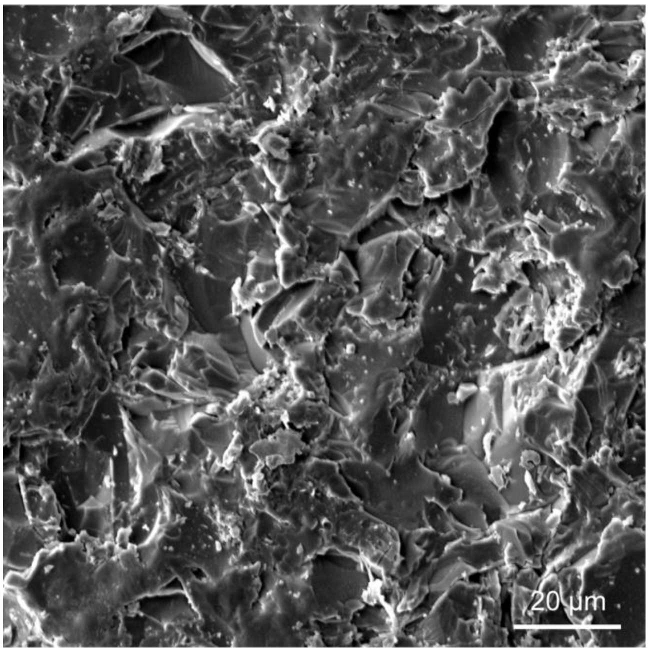

Figure S14. SEM image of the surface topography of the frosted glass slider.

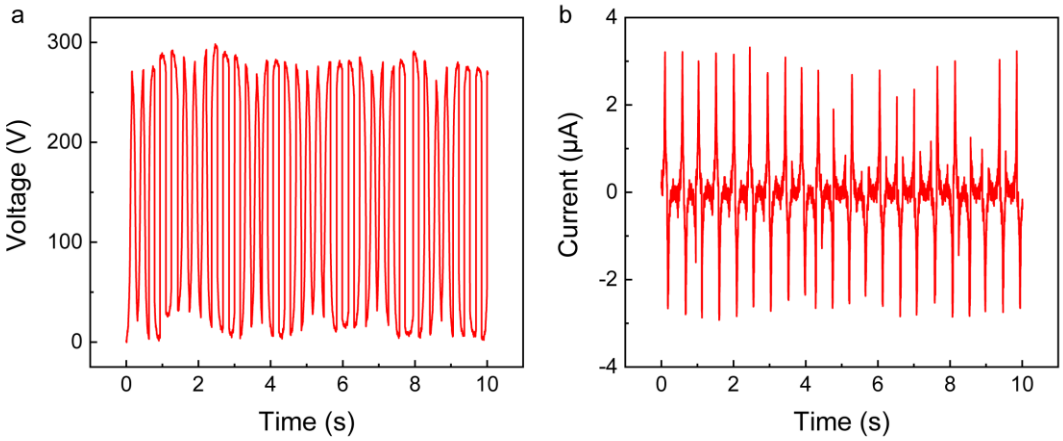

Figure S15. Output of the blue energy ball based on the DLC and glass pair. **a)** Open-circuit voltage and **b)** short-circuit current.
